# High Expression of *BCL11A* Predicts Poor Prognosis for Childhood *MLL*-r ALL

**DOI:** 10.3389/fonc.2021.755188

**Published:** 2021-12-06

**Authors:** Lu-Lu Wang, Dehong Yan, Xue Tang, Mengqi Zhang, Shilin Liu, Ying Wang, Min Zhang, Guichi Zhou, Tonghui Li, Feifei Jiang, Xiaowen Chen, Feiqiu Wen, Sixi Liu, Huirong Mai

**Affiliations:** ^1^ Department of Hematology and Oncology, Shenzhen Children’s Hospital, Shenzhen, China; ^2^ Guangdong Immune Cell Therapy Engineering and Technology Research Center, Center for Protein and Cell-Based Drugs, Institute of Biomedicine and Biotechnology, Shenzhen Institutes of Advanced Technology, Chinese Academy of Sciences, Shenzhen, China

**Keywords:** acute lymphoblastic leukemia, *MLL* rearrangement, *BCL11A*, differentially expressed gene, WGCNA

## Abstract

**Background:**

Despite much improvement in the treatment for acute lymphoblastic leukemia (ALL), childhood ALLs with *MLL*-rearrangement (*MLL*-r) still have inferior dismal prognosis. Thus, defining mechanisms underlying *MLL*-r ALL maintenance is critical for developing effective therapy.

**Methods:**

GSE13159 and GSE28497 were selected *via* the Oncomine website. Differentially expressed genes (DEGs) between *MLL*-r ALLs and normal samples were identified by R software. Next, functional enrichment analysis of these DEGs were carried out by Gene Ontology (GO), Kyoto Encyclopedia of Genes and Genomes (KEGG), Gene Set Enrichment Analysis (GSEA), and Search Tool for the Retrieval of Interacting Genes/Proteins (STRING). Then, the key hub genes and modules were identified by Weighted Gene Co-expression Network Analysis (WGCNA). Therapeutically Applicable Research to Generate Effective Treatments (TARGET) ALL (Phase I) of UCSC Xena analysis, qPCR, and Kaplan-Meier analysis were conducted for validating the expression of key hub genes from bone marrow cells of childhood ALL patients or ALL cell lines.

**Results:**

A total of 1,045 DEGs were identified from GSE13159 and GSE28497. Through GO, KEGG, GSEA, and STRING analysis, we demonstrated that *MLL*-r ALLs were upregulating “nucleosome assembly” and “B cell receptor signal pathway” genes or proteins. WGCNA analysis found 18 gene modules using hierarchical clustering between *MLL*-r ALLs and normal. The Venn diagram was used to filter the 98 hub genes found in the key module with the 1,045 DEGs. We identified 18 hub genes from this process, 9 of which were found to be correlated with *MLL*-r status, using the UCSC Xena analysis. By using qPCR, we validated these 9 hub key genes to be upregulated in the *MLL*-r ALLs (RS4;11 and SEM) compared to the non-*MLL*-r ALL (RCH-ACV) cell lines. Three of these genes, *BCL11A*, *GLT8D1* and NCBP2, were shown to be increased in *MLL*-r ALL patient bone marrows compared to the non-*MLL*-r ALL patient. Finally, Kaplan–Meier analysis indicated that childhood ALL patients with high *BCL11A* expression had significantly poor overall survival.

**Conclusion:**

These findings suggest that upregulated *BCL11A* gene expression in childhood ALLs may lead to *MLL*-r ALL development and *BCL11A* represents a new potential therapeutic target for childhood *MLL*-r ALL.

## Introduction

Acute lymphoblastic leukemia (ALL) is the most common childhood malignancy, which is characterized by recurrent chromosomal and molecular abnormalities of immature lymphoid cells (1). About 9% of adult and 3%–5% of children ALL cases harbor rearrangements of the mixed-lineage leukemia (*MLL*) gene (2, 3). Despite the much improvement in treatment for ALL, childhood ALL with *MLL*-rearrangement (*MLL*-r) still displays inferior prognosis attributed to hyperleukocytosis, aggressive form with early relapse, and central nervous system involvement (4–6), distinguishing itself from other leukemia subgroups.

*MLL*-r genes form fusions with more than 80 partner genes, of which the most common ones are AF4, AF9, ELL, and ENL (7). *MLL*-AF4 fusion represents the largest subgroup and is associated with a particular poor prognosis (8). Recently, much progress has been made in understanding the *MLL*-r leukemia process by using array-based technologies. A subset of dysregulated genes were found in *MLL*-r ALL, especially posterior *HOXA* genes and *MEIS1* genes (9, 10). Previous studies demonstrated that the *MLL* fusion proteins directly stimulated the histone methylation and target genes transcriptional elongation with the cooperation of *DOT1L*, *PAFc*, and *pTEFb*, leading to the persistent expression of genes that are important for the cell signaling, regulation of hematopoiesis, and transcription (11–13). Thus, exploring novel genes and pathways associated with *MLL*-r ALL may help to identify potential molecular mechanisms, diagnostic markers, and therapeutic targets in this special subgroup.

In this study, weighted gene co-expression network analysis (WGCNA) was first used to analyze the hub genes of *MLL*-r ALL samples mined from the Gene Expression Omnibus (GEO) database. The hub genes closely associated with *MLL*-r ALL were further confirmed in the data of Therapeutically Applicable Research to Generate Effective Treatments (TARGET) ALL (Phase I), ALL cell lines, and childhood ALL patients. We also evaluated the prognostic significance of hub gene expressions in the overall survival (OS) for childhood ALL patients. Our results provided the framework of co-expression gene modules of *MLL*-r ALL, which would be beneficial to the clinical diagnosis and treatment of childhood *MLL*-r ALL.

## Materials and Methods

### ONCOMINE Analysis

ONCOMINE database (https://www.oncomine.org) is an integrated data-mining platform that analyzes previously published or open-access cancer microarray data. Using the keywords “acute lymphoblastic leukemia” and “Cancer vs. Normal Analysis” for searching ALL databases, we identified two GEO Series (GSE, ID GSE13159 and GSE28497), which could be analyzed based on the same annotation platform. The GSE28497 was analyzed by the GPL96, while the GSE13159 was analyzed by GPL570. As described by the GEO website, all probe sets represented on the GPL96 are identically replicated on GPL570. Thus, we could analyze both GSEs by the same annotation platform GPL570.

### Data Collection and Preprocessing

GSE13159 contains 74 normal samples and 70 *MLL*-r ALL samples while GSE28497 includes 4 normal samples and 17 *MLL*-r ALL samples. Gene expression data from GSE13159 and GSE28497 were integrated manually to two parts, respectively, one containing 78 normal samples and another containing 87 leukemia samples for bioinformatics analysis. The combat function in the sva package was applied to remove the batch effects (**Supplementary Figure 1**) (14).

### Differentially Expressed Genes Screening

The robust multi-array average (RMA) in R software (version 3.6.5) was applied to explore the gene expression data (15). Differentially expressed genes (DEGs) between *MLL*-r ALL and normal cases were identified using the Bayesian method by the linear models for microarray expression data (LIMMA) package in R software (16). |Log_2_ fold change (FC)| > 1 and a *p*-value <0.01 were considered as threshold points. All the above operations were run with scripts in the R software Ggplot2 package was conducted to show the heatmap and volcano map.

### Protein–Protein Interaction Network Building

DEGs were taken into Search Tool for the Retrieval of Interacting Genes/Proteins (STRING) with the maximum number of interactors = 0 and a confidence score ≥ 0.4 as the cutoff criterion. Then, the result was analyzed by Cytoscape (17). Top 40 genes were screened using the plug-in CytoHubba in Cytoscape (18). CytoHubba provides a simple interface to analyze the node essentiality on the selected network with 11 scoring methods. Then, the bio-functional modules in the top 40 genes were explored using a plug-in MCODE in Cytoscape with a Node Score Cutoff of 0.2, a degree cutoff of 2, and a *k*-Core of 2. Then, the genes in the three modules were taken into the DAVID website. Gene ontology (GO) and The Kyoto Encyclopedia of Genes and Genomes (KEGG) enrichment analyses were carried out based on DAVID (http://david.ncifcrf.gov/summary.jsp). The significance threshold was *p* < 0.05.

### Gene Set Enrichment Analysis

Gene set enrichment analysis (GSEA) was conducted between *MLL*-r ALL and normal samples. Expression dataset from GSE13159 and GSE28497 was converted to the tab delimited gene cluster text (GCT) format following the operations according to the protocol (http://www.gsea-msigdb.org/gsea/). False discovery rate (FDR) < 0.05 and |normalized enrichment score (NES)| > 1 were regarded as the cutoff criteria.

### WGCNA

WGCNA package of R software was applied to uncover the correlation among genes. Firstly, gene expression data from GSE13159 and GSE28497 were input into R software to inspect good genes and samples. Sample clustering was used to detect outliers and match the samples with their characteristics. The soft thresholding power of *β* was set at 6 to ensure a scale-free network. The minimum number of module genes was set at 30. The hierarchical clustering dendrogram summarized the gene modules with different colors. Heatmap and topological overlap matrix (TOM) plot were used to visualize the module structure. The clinical traits were classified into two subtypes, “Normal” and “Leukemia”, for constructing module–trait relationships. For each expression profile, the gene significance (GS) and module membership (MM) were defined as the correlation value for each trait and each module eigengene, respectively. Hub genes are a class of highly connected genes, which have high connectivity with other genes in the same gene module and clinical trait.

### UCSC Xena Analysis

The gene expression data of TARGET ALL (Phase I) were analyzed by the University of California, Santa Cruz (UCSC) Xena (http://xena.ucsc.edu/). Hub gene expression levels were calculated between *MLL*-r and non-*MLL*-r ALL patients. The minimal residual disease (MRD) status on Day 8 and Day 29 of patients were also detected. The TARGET ALL (Phase I) project was obtained from patients enrolled on biology studies and clinical trials managed through the COG, POG 9906 (clinical trial for patients with newly diagnosed ALL between March 2000 and April 2003 that were defined as high risk for relapse). Patient samples for full characterization were chosen based on the following criteria: the disease onset at >9 years of age; did not have white blood cell count > 50,000/µl; did not express BCR/ABL fusion gene; were not known to be hypodiploid (DNA index > 0.95); and achieved remission (fewer than 5% blasts) following the standard two rounds of induction therapy. The primary patient samples were collected at diagnosis and gene expressions were analyzed following the protocol of Human Genome U133 Plus 2.0 Array (Affymetrix).

### Patients

Five childhood *MLL*-r ALL and 30 childhood non-*MLL*-r ALL bone marrow (BM) samples were procured from the newly diagnosed patients being treated in Shenzhen Children’s Hospital from 2018 to 2020. Informed consent was obtained from all patients or their parents. Experiments involving human materials were approved by the ethics committee of Shenzhen Children’s Hospital (approval number 202105102) and carried out according to the Declaration of Helsinki. Primary mononuclear cells were collected from the bone marrow samples by Ficoll density gradient centrifugation.

### Cell Culture

RS4;11 and RCH-ACV cells (ATCC) were maintained in RPMI 1640 with 10% fetal bovine serum (FBS) supplemented with 1% penicillin and streptomycin. SEM cells (ATCC) were maintained in IMDM with 10% FBS, 1% penicillin, and streptomycin. Cells were cultured in a cell incubator and maintained at 5% CO_2_ and 37°C.

### Quantitative Real-Time PCR

Total RNA of cells were reverse transcribed to cDNA with equal RNA volume (1 μg) using the PrimeScript™ RT Master Mix (Takara). Quantitative real-time PCR (qPCR) was performed using SYBR Green qPCR Master Mix (MedChem Express, USA) and detected on CFX96™ Real-Time System (BIO-RAD). Primer sequences for mRNA expression detection are shown in **Supplementary Table 1**. All experiments were repeated at least in triplicate. Gene expression levels were calculated relative to the housekeeping gene β-actin.

### Kaplan–Meier Analysis

The gene expression data of TARGET ALL (Phase I) were obtained from USCS Xena, and the clinical information was downloaded from the official website of the TARGET program (https://ocg.cancer.gov/programs/target). Finally, a total of 207 patients with gene expression data were analyzed by Kaplan–Meier analysis. The ggplot2 of R software was used to plot the Kaplan–Meier survival curve. The population was stratified by the upper 25th percentile (high) versus the lower 25th percentile (low) expression for key hub genes mRNA. The correlations between hub gene expression and OS, the hazard ratio (HR) with 95% confidence intervals, and *p*-value were determined by the Log-rank test. Log-rank test *p* < 0.05 was considered a significant difference. The results published here are in whole or part based on data generated by the TARGET (https://ocg.cancer.gov/programs/target) initiative, phs000218.

### Statistical Analysis

Student’s *t*-test of variance was used for comparing the statistical differences of mRNA expression of BM samples and ALL cell lines. All the analyses were two‐sided and *p* < 0.05 was considered to be a significant difference.

## Results

### Identification of DEGs in *MLL*-r ALL vs. Normal Samples

**Figure 1:**
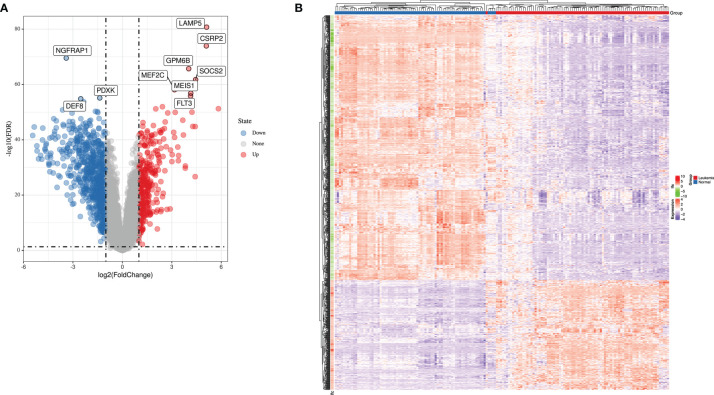
The expression profiles of *MLL*-r ALL. **(A)** Volcano map of differently expressed genes between *MLL*-r ALL and normal samples. **(B)** Heatmap of the DEGs according to the value of |logFC|.

Using the keywords “acute lymphoblastic leukemia” and “Cancer vs. Normal Analysis” for searching ALL databases in ONCOMINE website, we identified two GEO Series (GSE13159 and GSE28497) based on the same annotation platform. Gene expression data from GSE13159 and GSE28497 were integrated manually to two datasets (*MLL*-r ALL dataset containing 87 leukemia samples and normal samples dataset containing 78 normal samples, respectively). The gene expression data were analyzed using LIMMA package of R software and its extension packages. After removing the batch effect, we identified a total of 1,045 DEGs, 301 of which were upregulated genes while 744 were downregulated genes using adjusted |logFC| > 1 and *p-*value < 0.01 as cutoff threshold points. The most aberrantly expressed DEGs were marked in the volcano map, such as upregulated genes *LAMP5*, *CSRP2*, *GPM6B*, *SOCS2*, *MEF2C*, *MEIS1*, and *FLT3* and downregulated genes *NGFRAP1*, *PDXK*, and *DEF8* (**Figure 1A**). The heatmap of DEGs is shown in **Figure 1B**.

### GO, KEGG, GSEA, and STRING Pathway Analysis of DEGs from *MLL*-r ALL vs. Normal Samples

**Figure 2:**
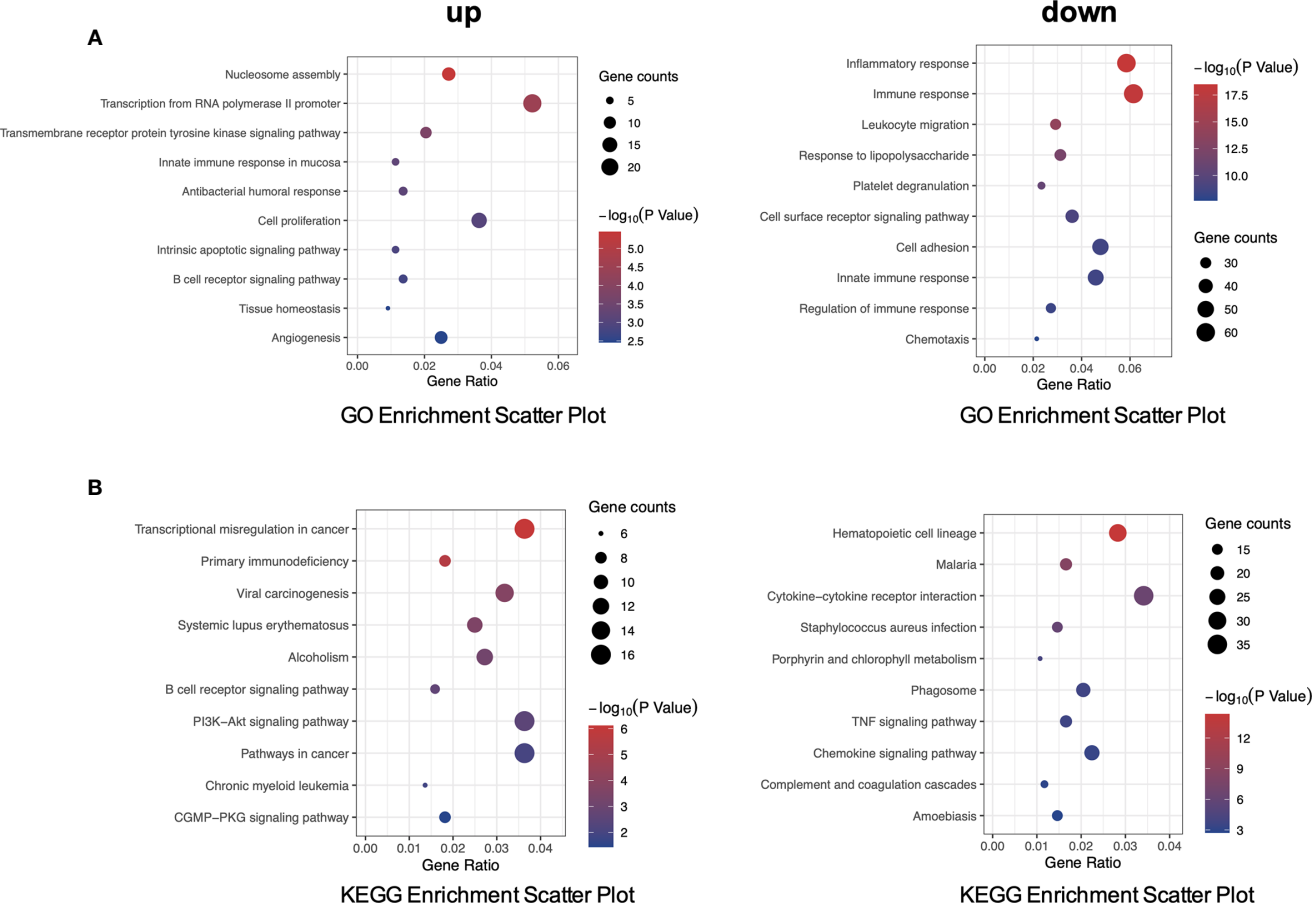
PPI network by STRING. **(A)** GO enrichment analysis of up- and downregulated DEGs. **(B)** KEGG pathway analysis of up- and downregulated DEGs.

**Figure 3:**
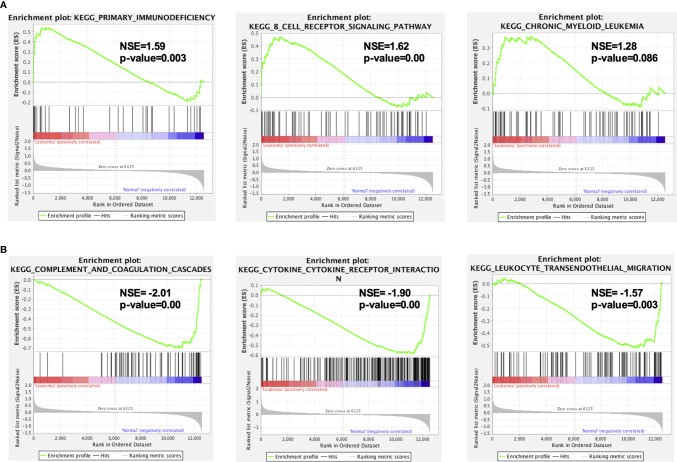
GSEA indicated the KEGG pathways in *MLL*-r ALL. **(A)** The gene sets enriched in *MLL*-r ALL samples were related to primary immunodeficiency and B-cell receptor signaling pathway (*p* < 0.05). **(B)** The gene sets enriched in normal samples were mainly related to complement and coagulation cascades, cytokine–cytokine receptor interaction, and leukocyte transendothelial migration (*p* < 0.05).

To gain insight into a more comprehensive knowledge of DEGs of *MLL*-r ALL vs. normal samples, the GO and KEGG pathway analysis of DEGs was carried out *via* DAVID online analysis tool. GO analysis showed that *MLL*-r ALLs were mainly upregulating “transcription from RNA polymerase II promoter, cell proliferation, nucleosome assembly” genes and downregulating “immune response, inflammatory response, cell adhesion” genes (**Figure 2A**). KEGG analysis showed upregulation in “transcriptional misregulation in cancer, pathways in cancer, PI3K-Akt signaling pathway” and downregulation in “cytokine-cytokine receptor interaction, hematopoietic cell lineage, chemokine signaling pathways” (**Figure 2B**). Additionally, GSEA showed *MLL*-r ALLs to be enriched in “primary immunodeficiency, B cell receptor signaling pathway” pathways (**Figure 3A**) and negatively correlated with “complement and coagulation cascades, cytokine-cytokine receptor interaction, leukocyte transendothelial migration” pathways (**Figure 3B**).

**Figure 4:**
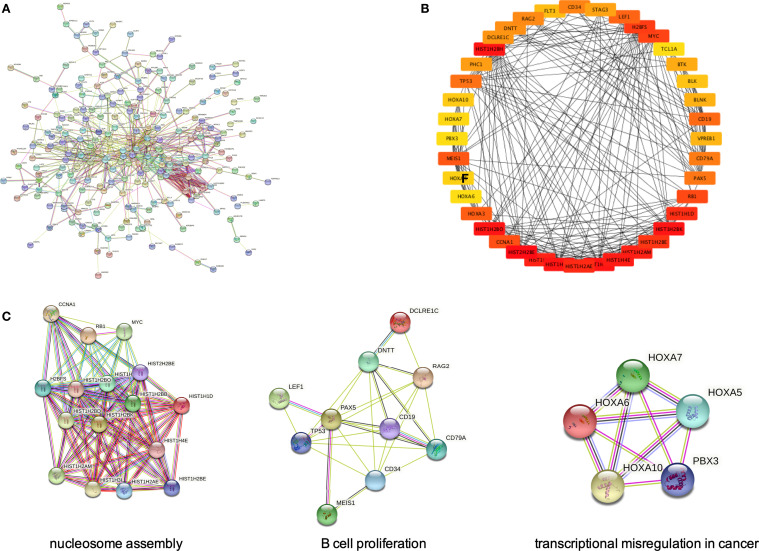
PPI networks of upregulated DEGs by STRING Search Tool. **(A)** The PPI network was constructed by STRING based on upregulated DEGs. **(B)** The 40 genes with highest CytoHubba scores were shown in a circle. The color scheme of each node from red to yellow represents the scores from high to low, respectively. **(C)** The genes with highest CytoHubba scores made up three critical subnetworks.

**Figure 5:**
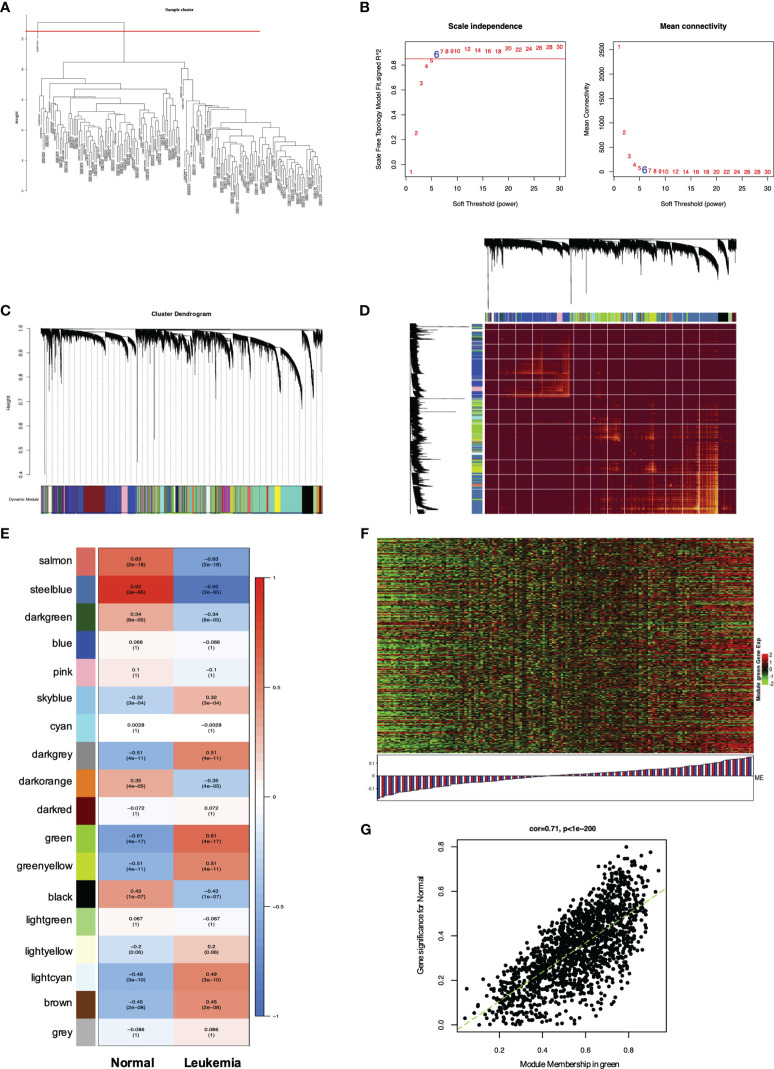
WGCNA of genes and modules in *MLL*-r ALL. **(A)** Cluster dendrogram of 165 samples. **(B)** Analysis of the scale-free fit index for various soft thresholding powers (*β*) and the mean connectivity for soft threshold power. **(C)** Hierarchical cluster tree showing adjacency modules based on topological overlap. **(D)** Heatmap of all DEGs formed a TOM plot to visualize the module structure. In the TOM plot, light yellow color represents low overlap and darker yellow color indicates higher overlap. Left side and the top side of the heatmap show the gene dendrogram and module assignment. **(E)** Correlation between each module and trait. Each row represents a module eigengene and each column represents a clinic trait. Each cell contains the correlation and *p*-value of each module. **(F)** Heatmap for the DEGs in the green module. **(G)** A scatter plot of eigengenes significance versus modular membership in the green module. Intramodular analysis of the genes found in the green module showed a high correlation with leukemia, with *p* < 1e-200 and correlation = 0.71.

Based on the STRING website, we built a protein–protein interaction network of up- and downregulated DEGs from *MLL*-r ALL vs. normal samples (**Figures 4A**, **5A**). In order to identify the key up- and downregulated DEGs between *MLL*-r ALL and normal samples, we adopted all the 11 methods in CytoHubba application, a plug-in of Cytoscape. *Via* calculation and analysis, the top 40 genes of up- and downregulated DEGs were identified separately (**Figure 4B** and **Supplementary Figure 2A**). Afterwards, the MCODE was employed to calculate the k-core of each gene and identify the critical networks in the top 40 genes. The upregulated gene with the highest k-core comprised three networks, which were involved in three important KEGG pathways: nucleosome assembly, B-cell proliferation, and transcriptional misregulation in cancer (**Figure 4C**). The top 40 downregulated DEGs with the highest k-score made up one important network, which was enriched in siderophore-dependent iron import into cell (**Supplementary Figure 2B**). Through GO, KEGG, GSEA, and STRING analysis, we demonstrated that *MLL*-r ALLs were upregulating “nucleosome assembly” and “B cell receptor signal pathway” genes or proteins and downregulating “complement and coagulation cascades” and “cytokine-cytokine receptor interaction” genes or proteins. These pathways were identified by at least two different biostatistics methods.

### Identification of Hub Genes and Modules of *MLL*-r ALL by WGCNA

WGCNA has been widely and successfully used to identify candidate biomarkers and therapeutic targets in various complicated diseases (19–21). To clarify the key modules and hub genes in *MLL*-r ALL, a total of 165 samples from GSE13159 and GSE28497 were input into R software for WGCNA, which was employed to uncover the highly correlated genes and the coexpression networks of *MLL*-r ALL. One GEO sample (GSM), GSM331691, was excluded from the analysis due to poor quality (**Figure 5A**). The power of *β* = 6 was set as the soft threshold for a scale-free network (**Figure 5B**). Subsequently, 18 gene modules were generated by the hierarchical clustering dendrogram, ranging in size from 2,947 (blue module) to 41 (gray module) (**Figure 5C**). After analyzing the interactions between the 18 modules, the TOM plot of a gene network was constructed with the corresponding hierarchical clustering dendrogram and the modules (**Figure 5D**). Based on the correlation between module eigengenes and clinical traits, the co-expressed genes were found primarily clustered in the green and steel blue modules. Among the modules, the green module was the most relevant with leukemia traits (**Figure 5E**). The heatmap of gene expression in the green module is shown in **Figure 5F**. We also explored the gene significance and module membership of the genes in the 18 modules. As shown in **Figure 5G**, the green module had significant correlations with leukemia trait. Eventually, 98 genes in the green module were selected for hub genes with a cutoff of intra-modular connectivity >0.85, demonstrating that these genes were not only the key components in module but also highly correlated with the leukemia trait. Taken together, we found 18 gene modules using hierarchical clustering between *MLL*-r ALLs and normal samples by WGCNA. The “Green” module was found to be most relevant with leukemia, 98 genes of which having the highest correlation with leukemia traits were selected for hub genes.

### Validation of Key Hub Genes in *MLL*-r ALL vs. non-*MLL*-r ALL Patients

**Figure 6:**
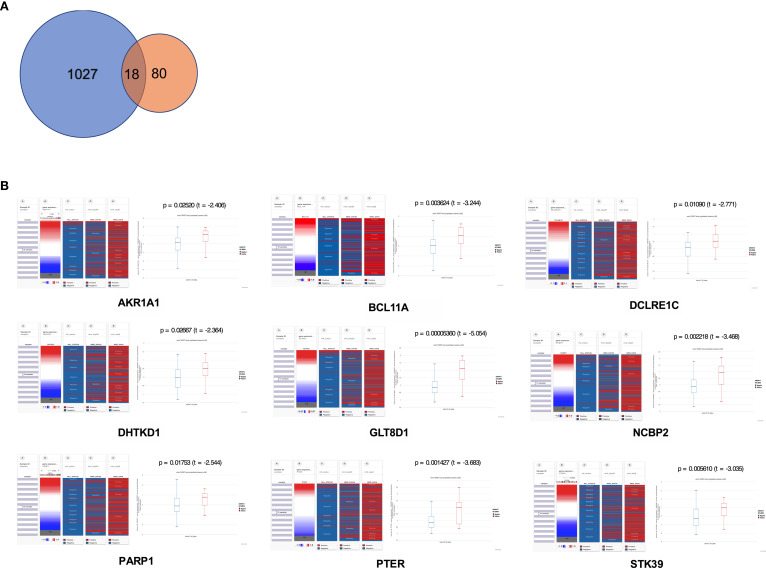
The expression level of key hub genes in childhood *MLL*-r ALL. **(A)** The Venn diagram showed hub genes identified from DEGs and WGCNA. **(B)** Expression of *AKR1A1*, *BCL11A*, *DCLRE1C*, *DHTKD1*, *GLT8D1*, *NCBP2*, *PARP1*, *PTER*, and *STK39* in *MLL*-r ALL was highly correlated with the *MLL* status in the TARGET ALL (Phase I) dataset (*p* < 0.05). The red color represents positive status and the blue color represents negative status of *MLL* fusion and MRD.

**Figure 7:**
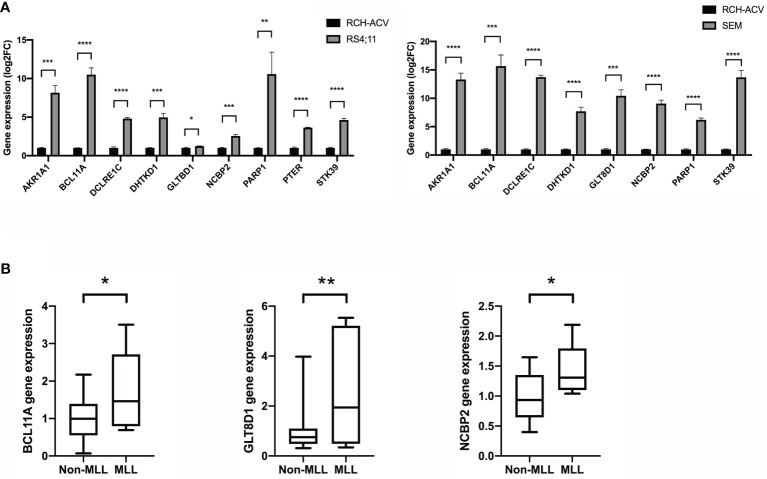
The gene expression level of key hub genes in *MLL*-r ALL and non-*MLL*-r ALL patients. **(A)** The mRNA expression of key hub genes were measured in RS4:11, SEM, and RCH-ACV cell lines. Data are normalized to *β*-actin and are representative of three independent experiments (*p < 0.05; **p < 0.01; ***p < 0.001; ****p < 0.0001). **(B)** The mRNA expression was detected in the BM of *MLL*-r ALL and non-*MLL*-r ALL patients. Results are expressed as a fold of the non-*MLL*-r ALL that is set as 1 (*p < 0.05; **p < 0.01).

To gain insight into the valuable hub genes in the green module, we used a Venn diagram to filter the 98 hub genes found in the “Green” module with the 1,045 DEGs from **Figure 1**. As shown in the Venn diagram, we identified 18 hub genes from this process (**Figure 6A**). Since the higher expression of 18 hub genes in *MLL*-r ALL is compared to the normal samples, we wondered which hub genes were also highly expressed in *MLL*-r ALL compared to non-*MLL*-r ALL. Thus, we further selected the TARGET ALL (Phase I) dataset to verify the differential expression of 18 hub genes between *MLL*-r and non-*MLL*-r ALL patients (22). Based on the results from UCSC Xena browser, nine key hub genes (*AKR1A1*, *BCL11A*, *DCLRE1C*, *DHTKD1*, *GLT8D1*, *NCBP2*, *PARP1*, *PTER*, and *STK39*) were found highly expressed in the *MLL*-positive samples compared to the *MLL*-negative samples (**Figure 6B**). Accordingly, to further determine the potential biomarkers for *MLL*-r ALL, we assessed hub gene expression in ALL cell lines and childhood ALL bone marrow samples. We found that the higher expression level of nine key hub genes were also observed in *MLL*-AF4 ALL cell lines (RS4;11 and SEM) compared to the non-*MLL*-r ALL cell line (RCH-ACV) (**Figure 7A**). Moreover, in our single clinical center, *MLL*-r ALLs had significantly higher mRNA expressions of *BCL11A*, *GLT8D1*, and *NCBP2* than non-*MLL*-r ALL patients (**Figure 7B**). However, there was no difference in *AKR1A1*, *DCLRE1C*, *DHTKD1*, *PARP1*, *PTER*, and *STK39* expression levels between *MLL*-r ALL and non-*MLL*-r ALL patients (not shown). These data suggest that upregulated *BCL11A*, *GLT8D1*, and *NCBP2* gene expression may be associated with the rearrangement of *MLL* in childhood ALL.

### High *BCL11A* Expression Correlated With Poor Overall Survival of ALL Patients

**Figure 8:**
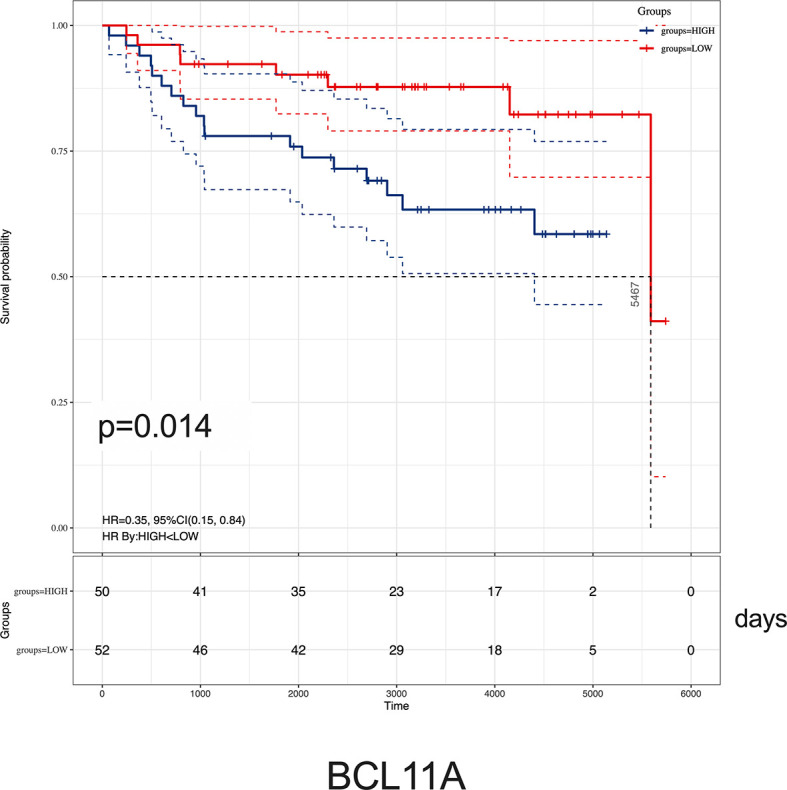
Kaplan–Meier analysis of *BCL11A* expression in childhood ALL. Survival cure comparing patients with high (blue) vs. low (red) *BCL11A* expression were plotted using a Log-rank test [HR = 0.35 (0.15–0.84), *p* = 0.014]. The unit of time is measured in days. The median survival time is 5,467 days.

Given the relationship between key hub genes and MLL status, we next examined the prognostic significance of hub genes in TARGET ALL (Phase I) datasets. We identified by Kaplan–Meier analysis that the high level of *BCL11A* mRNA expression was associated with inferior OS [HR = 0.35 (0.15–0.84), *p* = 0.014] (**Figure 8**). Nevertheless, there was no significant difference between the survival rate and the expression of *AKR1A1*, *DCLRE1C*, *DHTKD1*, *GLT8D1*, *NCBP2*, *PARP1*, *PTER*, and *STK39* (**Supplementary Figure 3**). Together, these results demonstrated that childhood ALL patients with high *BCL11A* expression had significantly poor OS, and *BCL11A* represents a new potential therapeutic target for pediatric *MLL*-r ALL.

## Discussion

Previous studies highlight the simple landscape of gene mutation, low DNA copy-number abnormalities, and limited window of genetic therapeutic targets for infant *MLL*-r ALLs (10, 23, 24). Transcriptional subversion and global gene expression alternation of *MLL*-r ALL play a greater role in this disease (25). Several microarray studies have already demonstrated the identification of DEGs related to distinct clinical and therapeutically relevant classes of leukemias (26, 27). However, the molecular alternation observed in *MLL*-r leukemia patients is rather complex and requires a more differentiated view. To gain a larger sample size, we combined two gene microarray expression datasets from GEO and conduct R software to analyze the DEGs in combined dataset.

As analyzed, in the upregulated gene or protein in *MLL*-r ALL samples, “B cell receptor signal pathway” was found to be significant in GO, KEGG pathway, and GSEA (**Figures 2**, **3A**), while “nucleosome assembly” was significant in GO and PPI network (**Figures 2**, **4C**). These results indicated that nucleosome assembly and B-cell receptor signal pathway were playing a core role in *MLL*-r ALLs. It is reported that *MLL* fusion genes enhance the process of transcriptional elongation by directly or indirectly binding to RNA polymerase II, leading to the alternation of whole genetic expression (28, 29). With the help of RNA polymerase II, *MLL* fusion genes drive the proliferation and self-renewal of immature hematopoietic cells by upregulating posterior *HOXA* genes and their cofactor *MEIS1* (30, 31). Similarly, GO analysis in our study demonstrated that the upregulated DEGs were significantly related with RNA polymerase II pathway (**Figure 2A**). *HOXA* and *MEIS1* were also identified in our PPI analysis (**Figure 4C**). Thus, the “transcription from RNA polymerase II” pathway may also be involved in the *MLL*-r ALL pathogenesis in this study.

WGCNA is widely used to explore the huge and complex relationships among genes across microarray or RNA sequence data. It provides a convenient and effective solution for screening core genes (32). Here, we first used WGCNA to explore the relationship of gene modules and clinical traits in *MLL*-r ALLs. As shown in the result, 18 upregulated genes were screened as hub genes by conducting WGCNA (**Figure 7A**), among which 9 key hub genes were confirmed to be highly associated with the *MLL*-r status in the TARGET ALL (Phase I) dataset (**Figure 7B**). In our childhood *MLL*-r ALL patients, *BCL11A*, *GLT8D1*, and *NCBP2* had significantly higher expression in *MLL*-r ALL patients than that in the non-*MLL*-r ALL patients. One limitation of this study is that there were only five *MLL*-r ALL BM patients for qPCR in the data analysis of our single center. Thus, further investigation is required to demonstrate the relationship of *MLL*-r ALL patients and DEGs with high-throughput sequencing for multi-center data analysis.

It is noteworthy to mention that *BCL11A*, a C2H2 zinc finger transcription factor, was initially identified as a chromosomal translocation oncogene in B-cell malignancies (33, 34). It modulated hemoglobin switching by directing γ-globin gene promoter repression (35). Recent studies uncovered that *BCL11A* was a critical oncogene in many other cancers like triple-negative breast cancer, lung squamous carcinoma, colorectal cancer, and laryngeal squamous cell carcinoma (36–39). In acute myeloid leukemia, high expression of *BCL11A* and *MDR1* was significantly associated with the poor prognosis (40). As analyzed using Kaplan–Meier, we reported that childhood ALL patients with high *BCL11A* expression showed inferior OS (**Figure 8**). Given the significant difference of *BCL11A* found by integrated analyses, we proposed that *BCL11A* could be considered as a potential target for ALL treatment and the molecular mechanisms study should be necessary to elucidate the role of *BCL11A* in *MLL*-r ALLs.

The high sensitivity of MRD has a profound influence on the treatment adjustment and the outcome of ALL patients during follow-up. MRD monitoring has been used to determine the ALL stratification in all childhood ALL. It was reported that MRD-positive status in Day 8 and Day 29 was associated with shorter event-free survival (EFS) in all risk groups (41). *MLL*-r ALL with positive MRD has significant worse EFS compared to those with negative MRD (42). To determine whether the poor prognosis that occurs with positive MRD is because of the hub gene expression, we examined the Day 8 and Day 29 MRD status of childhood ALL patients in the TARGET ALL (Phase I) dataset and their relationship with hub gene expression. However, there was no significant relationship between gene expression and MRD status (**Figure 6B**), neither in our single-center clinical data (not shown).

In the current study, we first integrated the information on DEGs through different bioinformatics analysis and identified that the nuclear assembly and B-cell receptor signaling pathway were the key pathways in *MLL*-r leukemia. Furthermore, we combined the results of WGCNA and DEGs, validated by clinical samples, and found that *BCL11A*, *GLT8D1*, and *NCBP2* were the key hub genes highly associated with the *MLL* status in childhood *MLL*-r ALL patients. Childhood ALL patients with high *BCL11A* expression had significantly poor OS. These findings suggest that upregulated *BCL11A* hub gene expression in childhood ALLs may lead to *MLL*-r ALL development and *BCL11A* represents a new potential therapeutic target for childhood *MLL*-r ALL.

## Data Availability Statement

Publicly available datasets were analyzed in this study. This data can be found here: https://www.ncbi.nlm.nih.gov/geo/query/acc.cgi?acc=GSE13159; https://www.ncbi.nlm.nih.gov/geo/query/acc.cgi?acc=GSE28497; https://ocg.cancer.gov/programs/target/resources.

## Ethics Statement

The studies involving human participants were reviewed and approved by the ethics committee of Shenzhen Children’s Hospital. Written informed consent was obtained from all patients or their parents.

## Author Contributions

L-LW and DY conceived the project, analyzed the datasets, and wrote the manuscript. XT, MQZ, and MZ performed the experiments. SLL, YW, GZ, TL, and FJ contributed analysis tools. XC, FW, SXL, and HM revised the manuscript and discussed the data. All authors contributed to the article and approved the submitted version.

## Conflict of Interest

The authors declare that the research was conducted in the absence of any commercial or financial relationships that could be construed as a potential conflict of interest.

## Publisher’s Note

All claims expressed in this article are solely those of the authors and do not necessarily represent those of their affiliated organizations, or those of the publisher, the editors and the reviewers. Any product that may be evaluated in this article, or claim that may be made by its manufacturer, is not guaranteed or endorsed by the publisher.
