# Supplementary material for: High Expression of BCL11A Predicts Poor Prognosis for Childhood MLL-r ALL
Source: Front Oncol. 2021 Dec 6;11:755188. doi: 10.3389/fonc.2021.755188 (PMC8685382; doi:10.3389/fonc.2021.755188)
Supplement: Supplementary file 1 [file DataSheet_1.docx]

Supplementary Material

| **Primer name** | **sequence(5' to 3')** |
| --- | --- |
| AKR1A1-F | ACTGGGCAGAAGATGCCTCTGATT |
| AKR1A1-R | TCAGGCTCATTGCCGTAGATAGCA |
| BCL11A-F | TTGCCCCAAACAGGAACACA |
| BCL11A-R | CGGGGCATATTCTGCACTCA |
| DCLRE1C-F | TCCCAGTCAACACACATAACA |
| DCLRE1C-R | GTCAGCTTTGTCCAAGGAAGTA |
| DHTKD1-F | GCATCGTCATCCTCCTTCCACATG |
| DHTKD1-R | CTCTTCCGCACTGTCACACATCTG |
| GLT8D1-F | GCGACGCTCTAGCGGTTA |
| GLT8D1-R | CGAGCACACTTGCCCTCT |
| NCBP2 -F | TTTCCAACATATAACGTACAGCTTTT |
| NCBP2 -R | CTACGTGGAGCTGAGCCAGT |
| PARP1-F | GCAGAGTATGCCAAGTCCAACAG |
| PARP1-R | ATCCACCTCATCGCCTTTTC |
| PTER-F | CGAGACACACAGACGTTGAAGAGG |
| PTER-R | CATCGGTAAGCTGCTCCACTGAC |
| STK39-F | ACAGTCCCTCTCTGTGCACG |
| STK39-R | ACGAGGTTCACGGCACAAGA |

**Supplementary Table 1.** Primers used for the RT-qPCR of hub genes.

**Supplementary Figure 1.** Data distribution of gene expression profiling data from GSE13159 and GSE28497. Before and after removing the batch effect.


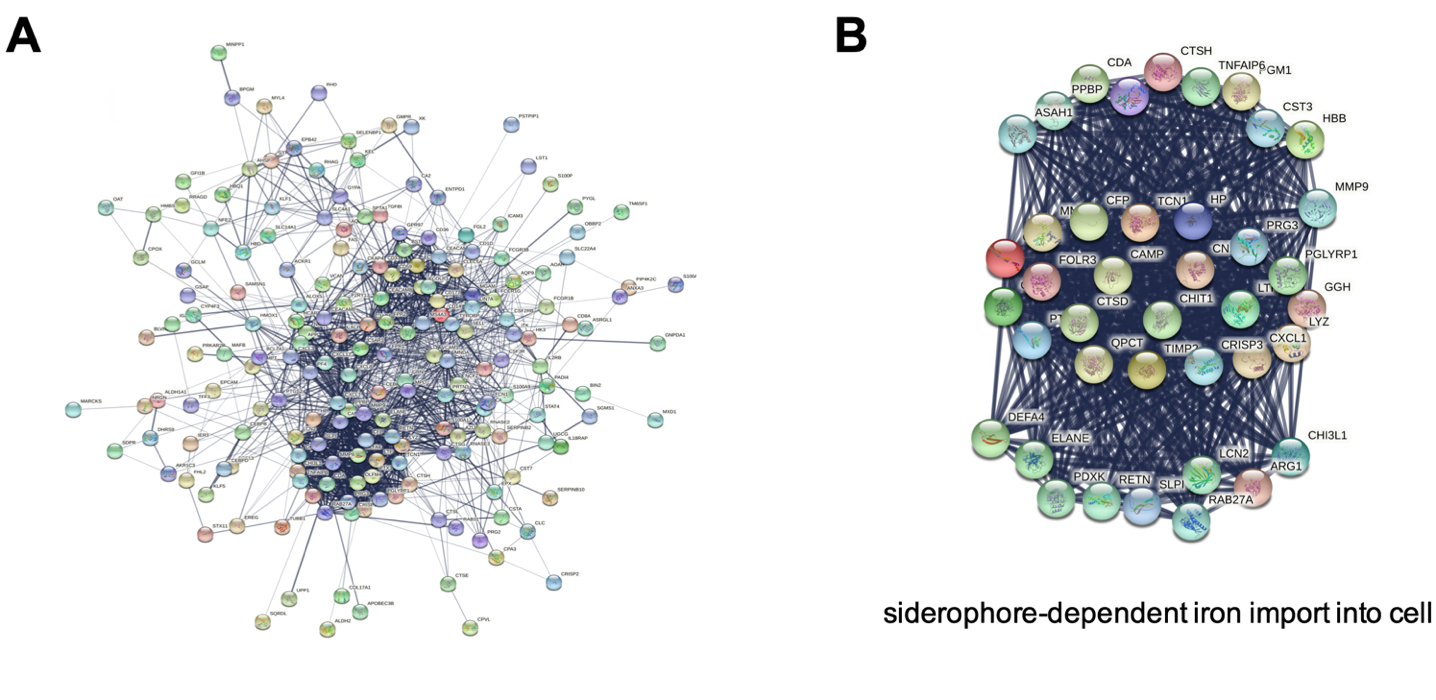


**Supplementary Figure 2. PPI networks** **of down-regulated DEGs by STRING Search Tool. (A)** The PPI network of down-regulated DEGs were constructed with the minimum required interaction score >0.4. **(B)** The down-regulated DEGs with highest k score made up 1 important network.

**
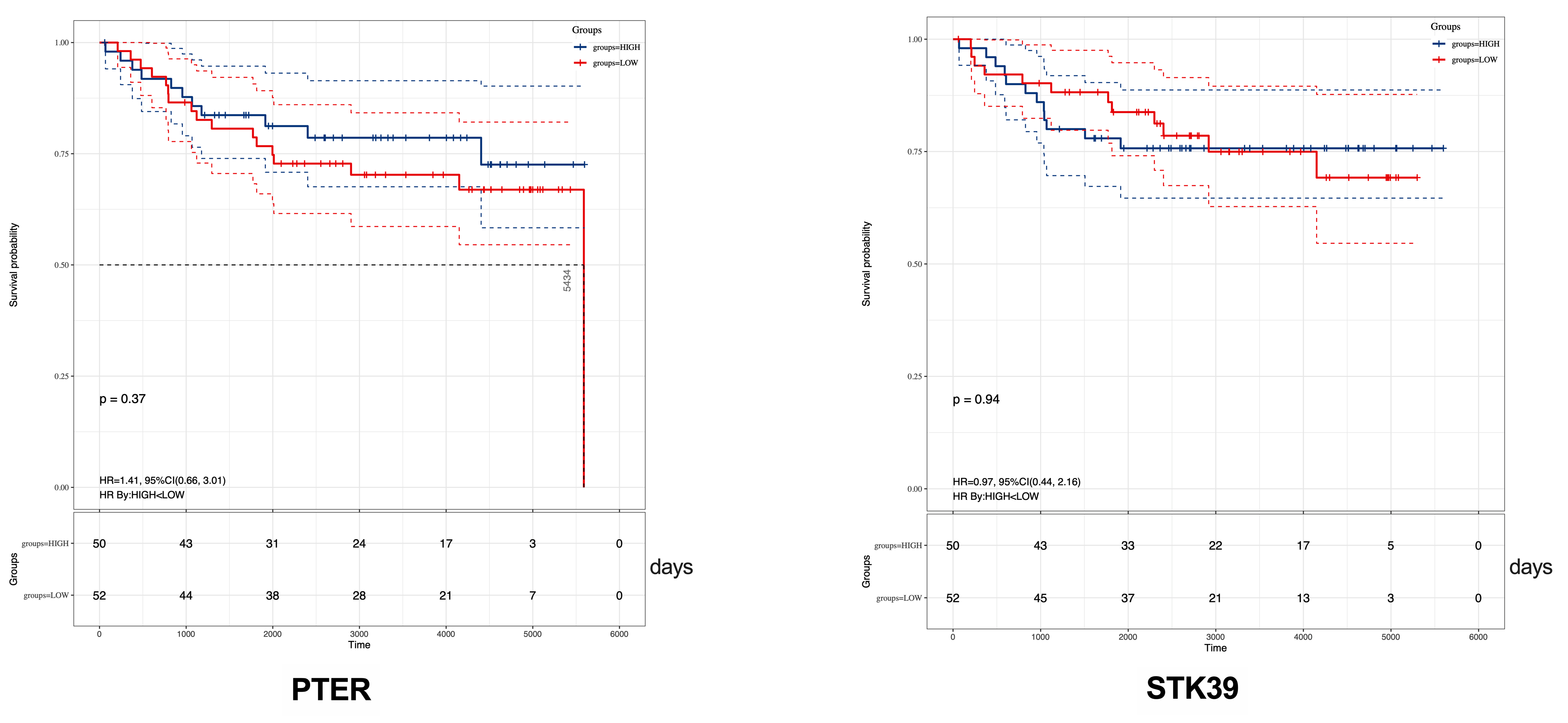
**
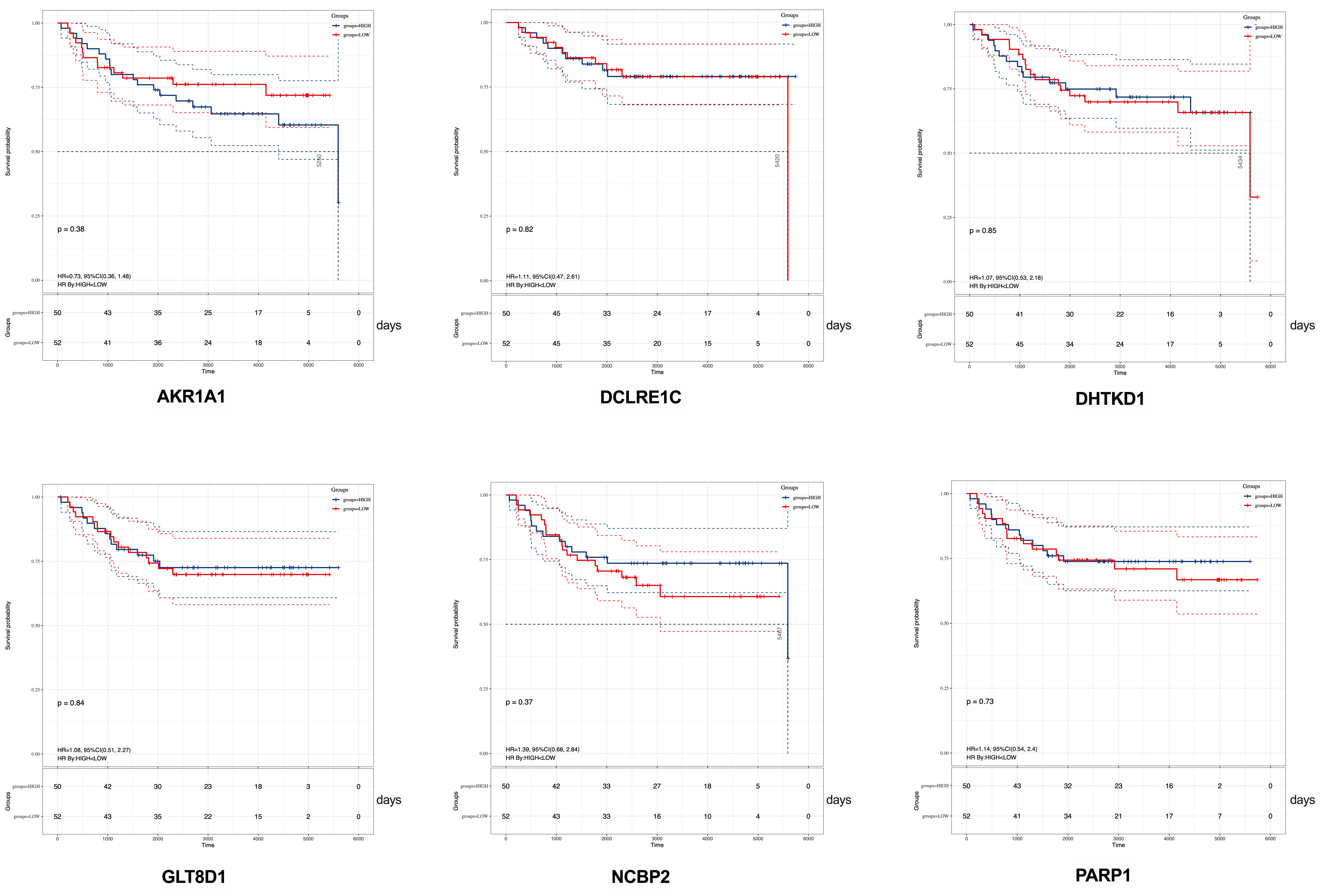


**Figure 3. Kaplan-Meier analysis of *AKR1A1*, *DCLRE1C*, *DHTKD1*, *GLT8D1*, *NCBP2, PARP1, PTER* and *STK39* in TARGET ALL (Phase I) dataset.** Survival cure comparing patients with high (blue) vs. low (red) of key hub gene expression were plotted using a log-rank test.
